# Conway's “Game of Life” and the Epigenetic Principle

**DOI:** 10.3389/fcimb.2016.00057

**Published:** 2016-06-14

**Authors:** Lorena Caballero, Bob Hodge, Sergio Hernandez

**Affiliations:** ^1^Centro de Ciencias de la Complejidad C3, Universidad Nacional Autónoma de MéxicoMexico City, Mexico; ^2^Institute for Culture and Society, Western Sydney UniversityParramatta, NSW, Australia

**Keywords:** game of life, epigenetics, computer simulations, fractality, Turing

## Abstract

Cellular automatons and computer simulation games are widely used as heuristic devices in biology, to explore implications and consequences of specific theories. Conway's Game of Life has been widely used for this purpose. This game was designed to explore the evolution of ecological communities. We apply it to other biological processes, including symbiopoiesis. We show that Conway's organization of rules reflects the epigenetic principle, that genetic action and developmental processes are inseparable dimensions of a single biological system, analogous to the integration processes in symbiopoiesis. We look for similarities and differences between two epigenetic models, by Turing and Edelman, as they are realized in Game of Life objects. We show the value of computer simulations to experiment with and propose generalizations of broader scope with novel testable predictions. We use the game to explore issues in symbiopoiesis and evo-devo, where we explore a fractal hypothesis: that self-similarity exists at different levels (cells, organisms, ecological communities) as a result of homologous interactions of two as processes modeled in the Game of Life

## Introduction

In recent times, computer simulations have played an increasingly important role in biology, in testing hypotheses and generating new ones. They provide judgment on the strengths of competing hypotheses, and generate unexpected or unsuspected possibilities for biologists to study and prove empirically. In this article we explore the merits for these purposes of a simulation game called “Life” by its creator, John Conway, “the Game of Life” by others. We see this second purpose, its “heuristic” or discovery function, as especially productive for biology. We show how this crucial function for practicing scientists can be found in the strategic use of versions that are usually dismissed by scientist as trivial and unserious.

Conway made connections with biology part of his purpose, bringing out “analogies with the rise, fall and alternations of a society of living organisms” (Gardner, 1970). This purpose explains the interest of this game for biologists, since it explicitly aims to model a basic process in biology, the evolution of ecological communities (see Caballero et al., 2014). Yet for heuristic purposes it is equally important to apply it to phenomena which were not part of the original intention. In that way we can test whether the game has a heuristic function, the capacity to develop new explanations which were not envisaged in the initial design. In the process we can understand better what deeper biological principles are being modeled in this simulation.

One interesting feature of this class of model is that it incorporated a general principle that later became a major factor in chaos theory, the idea of “deterministic chaos”: that is, the smallest number of rules which could generate an inherently unpredictable system. Lorenz (1995) and Eckmann and Ruelle (1985) proposed a three dimensional system as fitting this specification. Conway did not refer to this work but he implemented the principle, stating that “the rules should be such as to make the behavior of the population unpredictable.” He was not simply modeling any elemental biological system, he was modeling indeterminate chaos. In this respect he was adding a new requirement for complex biological models like those of Maturana et al. (1984), that they produce chaos (unlimited and unpredictable diversity) as well as complexity. We apply his model in the first place to epigenetic processes, which we understand in a broad sense, to refer to all mechanisms which act on the realizations of genetic action, not just to heritable DNA-modifications such as methylation. Epigenetics is now a broadly accepted aspect of genetics. In the 1950s and 1960s as proposed by Waddington it was seen as a competitor to genetics. However, Waddington's concept was designed as “a true synthesis between developmental processes and genetic action, which together bring the organism into being” (Van Speybroeck et al., 2002, p. 33).

Epigenetics in this context are all those factors involved in the regulation of DNA that do not involve changes in the sequence (Waddington, 1962; Jablonka and Lamb, 2015). The information encoded in the DNA of cells is the same for each cell of an organism. All cells and tissues of an organism arise from a primordial cell. Throughout development these acquire identities involving individual differentiation. Thus, we see large divergences between different cell types, which have specific functions. In the development from cells to complete organisms, there are important differences in what we call individuality. This individuality results from a series of informational factors formed by the genome and the epigenome, which is in feedback with environmental stimuli from the cellular level to the ecosystem.

There are many mechanisms implicated in epigenetic regulation. These include the marking of the DNA by various chemical groups that are bonded to the bases of the DNA; genomic imprinting; protein histone modification; regulatory ncRNAs (non-codifying RNAs); epigenetic mark maintenance; environmental effects (Inbar-Feigenberg et al., 2013); and the conditions of matter and physical aspects that contribute to the development of cellular systems (Caballero et al., 2012).

Environmental factors can alter epigenetic marks, impacting on the development of embryos and also affecting at least the next generation. This transgenerational effect of environmental conditions is a major focus of interest. It is still little-known, but it obviously opens up an interesting path in understanding the relationship of living beings with their environment, and therefore development and evolution (Jablonka et al., 2014; Lillycrop and Burdge, 2014; Skinner, 2014). If we want to model and explain the complexity of dynamic biological systems we need to recognize the importance of the epigenetic dimension, fundamental in constructing biological realities.

In this article we look especially at two seminal works in the development of mathematical epigenetic models, connecting them with Game of Life projections in order to develop a generative matrix for thinking about the foundations of epigenetic theory. Alan Mathison Turing is best known as a father of computing, but his model for morphogenesis (1952), often named the Reaction-Diffusion model, has proved an influential mathematical model for epigenetics (Turing, 1952). We look closely at his original proposal, which has been as hard to interpret as it has been influential. We also use a later application of his ideas specifically to biology, the Oster-Murray mechanochemical model (Oster et al., 1983; Murray et al., 1988) to help bring out some implications of Turing's ideas. As a complementary perspective we use Edelman's concept of topobiology (1988), again connected with the Game of Life.

## Conway's “game of life” and its biological referents.

GoL was described by Conway as a board game (1970) for zero or one player, but from the beginning it was played out on a computer format, in a program written by Michael Guy and Stephen Bourne. Conway said that without this format some discoveries about the game would have been difficult to make (Gardner, 1970).

Distinct from the Guy-Bourne program there is a range of commercial games, among them a package named “Golly,” (Rendell, 2011) which we also explore in this article. Conway presented his concept as a board game, to be played with two kinds of counter of different colors, e.g., black and white, played on a large grid. There are three main rules as stated in his description of the game:

Survivals. Every counter with two or three neighboring counters survives for the next generation.

Deaths. Each counter with four or more neighbors dies (is removed) from overpopulation. Every counter with one neighbor or none dies from isolation.

Births. Each empty cell adjacent to exactly three neighbors – no more, no fewer—is a birth cell. A counter is placed on it at the next move (Gardner, 1970).

These rules only begin to act after a set of counters has come to exist, in numbers and configurations that come from a decision process that is outside Conway's rules. In the game they come from the player, making decisions about counters and their positions. In computer versions of the game these decisions are made by algorithms. In both cases this second implicit set of rules can be called “existential,” since they determine both the fact and the configurations of existence. In biological terms, existential rules correspond to genetic rules. Conway's three spatial rules correspond to epigenetic rules.

As is a property of complex systems, the operation of these rules produces emergent new forms, with properties that are not predictable from the initial conditions. These new forms and properties do not involve changes in initial conditions, since the counters are unchanged, but in the emergent forms, the configurations that emerge, and their properties. They produce a wide variety of individual forms from a simple primordial origin

**Figure 1:**
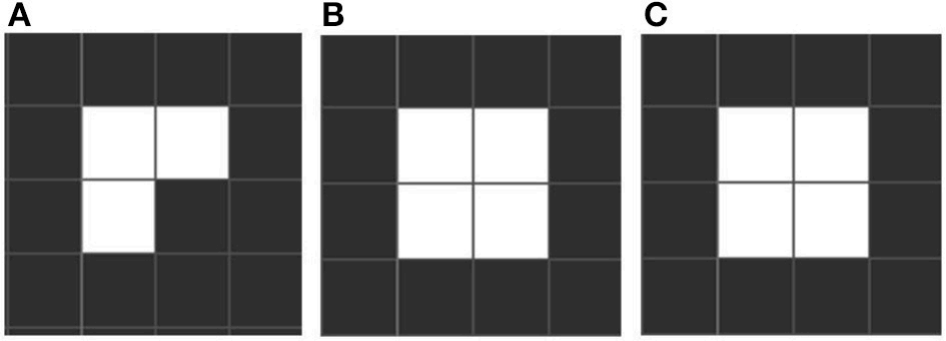
**An example of a still life**. The patter stabilizes into a fixed form. Still life forms illustrate the important biological point, that absence of change is something to explain, and it may be due to relations surrounding a given element that does not change, rather than being inherent in the element itself. **(A)** If having a configuration as showed then the rules imply that a new cell must come to live in the following iteration. **(B)** Four cells fixed iteration. **(C)** The configuration remains stable if it is not disturbed.

**Figure 2:**
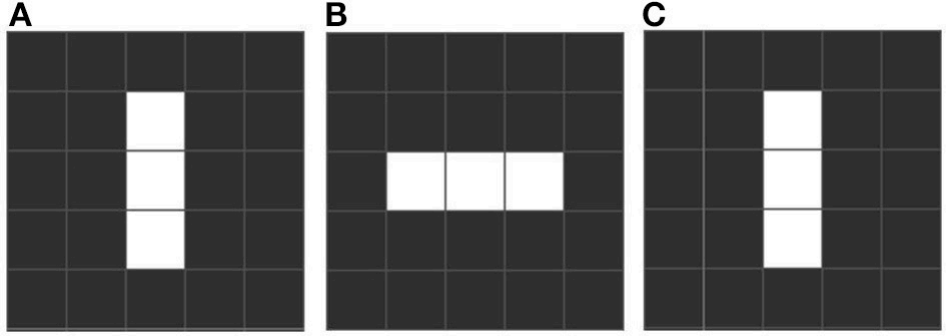
**An example of a blinker, which is a pattern that oscillates with a fixed period, that is, after n iterations the pattern returns to a previously visited state**. In the example this pattern has period 2. Rhythmic oscillations are common in biology, as in patterns of enervation. **(A)** First stated with three cells. **(B)** After one iteration the upper and lower cells die but the far left and right start to live. **(C)** Following the rules the blinker returns to the first iteration.

Conway identifies three distinctive emergent forms. “Still life” configurations are stable over many iterations, like a block of 4 adjacent squares (see Figure 1). “Oscillators” are stable over a cycle, returning to an initial state. Such cycles can be short (e.g., “blinkers,” Figure 2) or very long. Finally there is a rare, small set we call “movers,” which include “gliders,” which move across the grid. These emergent forms are exciting and remarkable, since oscillation and motion are two properties of living forms that were not part of the content of any of the rules, existential or conditional. See Table 1 which includes the equivalence between elements of the game of life and its related biological elements.

**Table 1:** **Equivalence between terms in Conway and Biology**.

Conway	Biology
Counter	Biological unit: gene, cell, organism, species
Survivals	reproduction, replication
Neighboring	environment, milieu
Rules	genetic, (realizing features encoded in genes) epigenetic (modifying expression of genes)

The output of the full set of rules combined can be seen as equivalent to phenotypes, the objects on which the processes of natural selection act. Conway uses the terms “overpopulation” and “isolation” from ecology, and he seems to be referencing evolutionary processes. However, he is modeling a generic situation, which can be found in many biological systems. He proposes a device which can produce greater diversity with fewer rules if those rules are organized in two sets: one set as in genetics, where elements are produced and replicated, the other as in epigenetics, where the output of those rules and processes in an epigenetic landscape produces a wide range of stable and novel forms, while restricting or eliminating many possible forms.

Conway's rules could also be applied to the organization of a holobiont in symbiopoietic theory, where the microbiome describes the environment that conditions the genetic outputs of the dominant organism (Zilber-Rosenberg and Rosenberg, 2008).

Conway's Game contributed to the theory of cellular automatons, a fertile theory in computing sciences associated with John von Neumann. Conway's version of this theory is often seen as a decisive vindication of it, making the theory simpler and easier to apply. At the same time, Conway's cellular automatons were different from Von Neumann's in some fundamental respects which are highly relevant to biological applications.

Von Neumann (1962), Neumann and Burks (1966) used the principle of cellular automatons to describe the instructions for what he called a Universal Constructor, whose purpose was to reproduce the capacity that for many people defines life, to be able to replicate itself: autopoiesis, in terms of Maturana and Varela (1987). The machine he proposed consisted of one part that is the functional structure to be replicated, a second part that copies the set of instructions, and a third part that inserts copies of instructions into the new structure.

Von Neumann's theory of the structure of a Universal Constructor predated Watson and Crick's decoding of DNA (Watson and Crick, 1953). Von Neumann's model is similar to their model of the replication of DNA. His Universal Constructor form of cellular automatons can be seen as a model for genetic processes.

This comparison brings out the originality and difference of Conway's proposal. Conway's cellular automatons model a double system, with some rules producing gene-like structures, while others are like epigenetic rules acting on those genetic structures. Variety and novelty characterize life just as much as does self-replication. The two properties, self-replication and variety, are both important, and adequate theories of life need to explain both.

In this comparison, cellular automatons drive both processes. This suggests that they may be related to some deep properties of life itself. Conway contributed to this line of research by applying the cellular automaton principle to epigenetic-like processes as well as to genetic processes, in a double structure that includes both genetic and epigenetic actions.

## Models in science

The work of Turing plays a key role in the argument of the paper. On the one hand he is often called a father of computers, in which capacity he influenced Von Neumann and Conway, and provided some illuminating ideas on computers and how they can be best used in fundamental scientific research. On the other hand he wrote an important article exemplifying the uses of mathematical biology, specifically applied to models of epigenetics. In this article we look at both aspects of his work.

Turing began his major article on epigenetics by reflecting on his use of mathematical models in biology: “This model will be a simplification and an idealization, and consequently a falsification” (1952, 37). This made modest claims for the real-life relevance of his biological mathematics. In practice, the connection was even weaker in some respects than this claim implied. He presented approximate mathematical solutions to aspects of the processes and mechanisms he included within his model. He did not provide equations for his morphogenetic model, only solutions for aspects of the problem. In effect he provided an overarching model which was not in itself expressed in mathematical form, which however organized a large, open-ended set of specific models, each with one or more associated mathematical model and mathematical equations.

In the conclusion he reflected on the role that digital computing might play, in contrast to the mathematical modeling he. He noted that using mathematics as he did “one cannot hope to have any very embracing theory of such processes, beyond the statement of the equation” (p72). In this situation he recommended a complementary use of digital computers, noting that in this way “it is not so necessary to make simplifying assumptions as it is when doing a more theoretical type of analysis.” In this case he added “It might be possible to take the mechanical aspects of the problem into account as well as the chemical” (p. 72).

It is important to recognize that these comments, which seem negative and pessimistic, were made by a pioneer in both mathematical biology and computing. He wanted to build a better basis for these two related aspects of theoretical biology by being aware of their limitations at that point in science.

Mathematical biology has developed substantially in the 50 years since Turing's article, in which Turing's work has had a significant place. In Murray's authoritative summary of the field “mathematics is required to bridge the gap between the level on which most of our knowledge is accumulating (in developmental biology it is cellular and below) and the macroscopic level of the patterns we see…The goal is to develop models which capture the essence of various interactions, allowing their outcomes to be more fully understood” (Murray, 2002).

In Murray's picture and Turing's practice we can identify a number of key distinctions affecting the way mathematics, models and computer simulations can be used in biology. Specific knowledge may accumulate in particular areas about particular phenomena, but that will fall short of full understanding without reference to models of mechanisms, especially as expressed in mathematical forms. Yet these models are likely to distort that reality, and mathematics is likely to make the models more distorted though more powerful.

In this situation, computer modeling can play a complementary role. In Turing's case he saw this new terrain as a possible site for including more assumptions, though in a simplified form. We apply this principle to GoL, when used as a research instrument as we do in this article, to explain how this artificial game can produce a model that explains some patterns and outcomes which are not so evident from other models and other mathematics.

## Epigenetic models

In this section we look at two mathematical models for epigenetic processes, and compare them to GoL.

We begin with Turing's model. In spite of its fame it is a difficult article which has proved hard to interpret. Turing's title refers to the “chemical basis” of morphogenesis, and later he states a concern with processes of “reaction” and “diffusion.” Based on this, his model has been referred to as a “Reaction-Diffusion” model. However, in his first statement of his model he describes four factors in it. One is “chemical reactions,” and another is the diffusion of the chemical substances. However, there are two others, both mechanical, one involving Newtonian laws of motion, the other involving various sources of stress.

Given this, we believe that Murray was correct to see Turing's full model as mechanicochemical, including Reaction-Diffusion processes as one instance among the full set of Turing devices (Murray and Oster, 1984). As we said in the previous section, Turing himself saw computers as a way of incorporating the range of factors, mechanical and chemical, into a single simplified but still comprehensive and complex model. That is the option we explore in the following.

Turing's work resonates with GoL in four ways:

1. Turing's concept of “morphogens” (literally “form producers”) includes genes and other factors, including Waddington's “evocators,” hormones and even pigments. That is, the term refers to both genetic and epigenetic factors affecting form. Both kinds of rule in GoL, existential and conditional, are morphogens in this sense.
2. Turing assumes that the function of genes is purely catalytic, that they persist unchanged after producing chains of effects. In GoL there is a common basic pattern, illustrated by what Conway called a blinker, which can be understood as involving catalysts. The blinker begins with a row of three cells. Following the rules, in the first iteration two of the three cells dies on either side of the central cell, but two new cells grow above and below the central cell. In the next iteration, the two new cells have died, but two new ones have been born, where there were two in the first iteration. In this process C continues unchanged, but without its presence the system would simply have died.
3. Turing examines a simplified system with only two morphogens, X and Y, though he also looks at the more complex case of 3 or more morphogens. Conway has two types of counter, black and white, but they do not correspond closely to Turing's. Black counters on their own mark all viable cells in one iteration or move. If a configuration leads to the death of a cell, then that is marked by a second black counter placed on it. White counters mark the site of a birth cell, which will be replaced by a normal black one in the next move. This means that black has two meanings. On its own it signifies a normal cell. Added to a normal cell it signifies the death of that cell. Conversely, white counters added to a non-cell signify that that cell will be alive for the next iteration, when it will be marked by a black counter.
  White and black in GoL do not signify life and death, activation and inhibition, but they do mark the outcomes of the two processes, which are fundamental in Turing's model. Turing proposes a series of reactions acting on his proposed morphogens X and Y, some of which produce X and Y, some of which destroy X and Y, and some which convert X into Y. This play of activation and inhibition is a basic property of Turing's model, so much so that some later scientists have seen it as the defining characteristic of this class of models. The function of producing life and death, activation and inhibition, is also fundamental in GoL, where it is carried by the conditions, which are like epigenetic properties of the system.
4. Turing shifts his attention from macrostructures of this class of system to local instances and patterns, which he subjects to precise mathematical analysis. One such local structure is a ring of similar cells. Turing analyses the operation of 2 or three morphogens in this kind of system. He identifies two main cases, which he calls the stationary and the oscillatory. He then further subdivides these two categories in terms of wave-length: extremely long and extremely short, and finite wave-lengths.
  This basic typology has interesting connections with Conway's typology of products of his processes. One he called “still life,” a stable form which once established repeats itself endlessly. This is like Turing's “stationary” case. A more interesting similarity is Conway's oscillatory figures, like the “blinker.” This is a highly surprising outcome, an emergent property of his game. It is also fundamental to Turing's model, where it only emerges in systems of 3 or more morphogens.
  Conway's oscillators are sometimes short, like the period-2 blinker above. But sometimes periodicity only emerges after many moves. In one case Conway reports an oscillator of 173 moves. Again this is a remarkable discovery, not obviously following from the initial conditions of the game.
  Conway treated all oscillators as in the same category, but Turing distinguished between very long and very short wave-lengths. Turing's distinction may be fruitfully applied to GoL games.
  Even more surprising and interesting among Conway's forms is a rare set of shapes which seem to move, bodies he calls “gliders” and “spaceships.” Although he discovered only 4 such forms, the existence of any of them is spectacular and unexpected. Movement is a property of life, along with self-maintenance, self-reproduction and functional death. Conway's program shows how all these properties can be products not premises of a biological system.
  Putting Turing and Conway together, we can see the value of seeing oscillation and movement alike as properties of a single epigenetic system, with significant differences in this continuum introduced by the factors Turing emphasizes: complexity (number of interacting morphogens) and wave-length (scale of periodicity). Perhaps there is a natural discontinuity in Conway's products between short and long-term oscillators, and between oscillators and movers.
  We complement Turing's model with the ideas of Edelman on topobiology (Edelman, 1993). Turing was not a biologist, and admitted himself that he did not know how his models would connect with the complex realities of biology then and now. In a sense the present article can be seen as a response to his request to biologists to embed his model more strongly in biological realities.

Edelman's work was firmly in the then-growing field of epigenetic theory. The term *topo*- referred to the importance of place and other spatial relationships, including the Newtonian and mechanical processes envisaged by Turing. Edelman [p. 17] proposed 5 primary processes involved in development, three of which he called “driving processes” and two he called “regulatory processes.” This division corresponds loosely to the distinction between genetic and epigenetic processes, but in Edelman's theory all are epigenetic. The driving processes are also milieu-dependent.

We reframe these processes in terms of GoL:

1. Cell division. GoL has a milieu-dependent version of this in its basic rules, where new cells are produced not exactly by replicating cells but by establishing spatial conditions under which the new cells (which are all identical, in GoL) will appear.
2. Cell death. Edelman's insistence on the importance of this as a driver of development is innovative in terms of biological theory. It is reflected in GoL's second constitutive rule, which regularly produces death. It corresponds in evolutionary theory to the role of selection, where the fit survive but the rest are eliminated. In epigenetic processes as they are currently understood, the process of switching genes off is as important as switching them on.
3. Cell movement. Although cells were known to move in the development process, it needs a topobiological framework to recognize the process. It is because cells move into different places that they undergo the range of mechanical processes that they do. Similarly, evolutionary theory does not usually emphasize the mobility of niches. Yet niches are dynamic systems, requiring and affecting constant change by organisms and ecosystems alike. Movement is a rare but remarkable and life-like property of GoL automatons. In Edelman's theory this property is essential for development.
4. Cell adhesion. This is one of Edelman's regulatory processes. It is not prominent in GoL, but it can be understood in terms of one GoL form:
  In this diagram, the first stage consists of three squares. Three squares are a vulnerable form in GoL, and indeed this is the only three-square structure which does not die. In this case, the configuration produces a new cell which binds the three into a tightly bonded structure which holds all members together over all later iterations. This form, which corresponds to Turing's stable form, may seem a minority output of the GoL, but along with blinkers it is the simplest form that survives destruction by epigenetic processes. Both are very common as products. A hypothesis that this analysis suggests is that a few very simple forms that persevere strongly may be in some respects elemental.
5. Differentiation and induction. By this Edelman refers to complex processes which may be interpreted as more than one process. In connecting this to GoL forms, the important factor here is that these processes operate with cell-collectives, not just individual cells. This is a crucial fact about developmental processes, but it is not easily covered in GoL terms. What it corresponds to is the relationship of large collectivities of cells interacting with each other.
6. Related to this point, Edelman emphasizes a framing condition of the topobiology of development, that the behavior of cells is very different in terms of time (stage in the process) and space (where the developing organism is). If this was modeled in GoL it would require some counter which would change rules of the game in response to a given number of iterations. This is a fundamental problem for epigenetic theories of development and morphogenesis. For instance, Turing is mainly concerned with relatively late stages of morphogenesis, where forms and organs are forming in an already existing organism, while Edelman is more concerned with embryology. Somehow the same processes must be at work in these very different stages, involving different balances of genetic and epigenetic factors. GoL is not at the moment suited to model such different outcomes.

## Computer games as laboratories

Thus, far we have looked at two conditions under which GoL can be mobilized as a heuristic instrument, the original board game and the related computer program of Guy and Bourne. To these we add Golly, an open-source cross-platform application developed primarily by Trevorrow and Rokicki (2005). All three formats would usually be seen as variants of a single set of principles. However, if we apply the reasoning behind the Epigenetic principle we can see the three formats as behaving like three different epigenetic landscapes, interacting with the core principles to produce sometimes novel outcomes. In this section we look at the heuristic value of the software program Golly.

Golly's primary purpose is to produce Cellular Automaton versions of GoL, with cells up to 256 states. It can also produce Von Neumann's 29-state cellular automaton, and other cellular automatons. We used it experimentally to explore some aspects of GoL which were not obvious in the other two formats.

**Figure 3:**
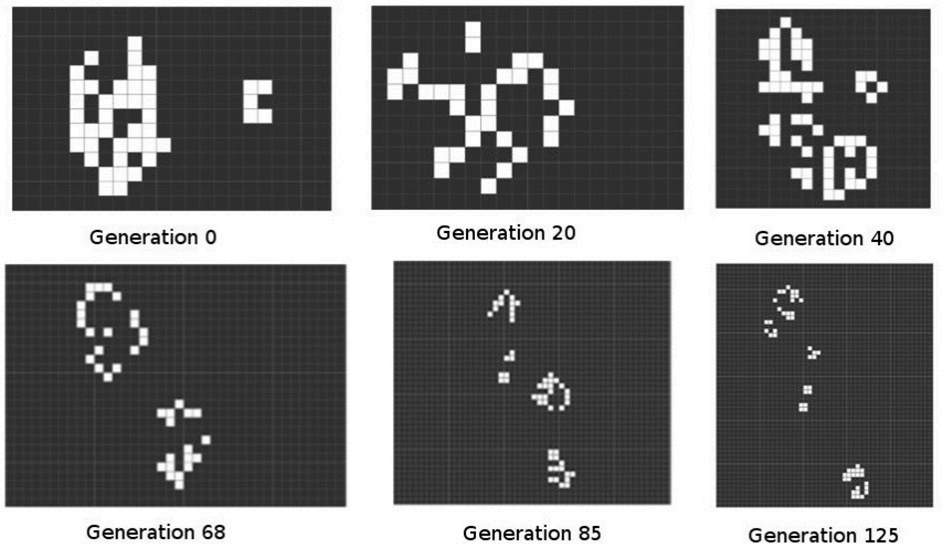
**Free evolution of a random initial condition**. The example shows some selected generations of 125 possible iterations. This figure was produced by the Golly program, and illustrates the systematic yet unpredictable outputs of this class of system.

1. The starting point conditions for GoL are not clearly specified by Conway, who left key decisions to his players. Golly has an option for a player to scribble a shape. It is easy to scribble a shape which covers many squares, so this starting point typically produces many more counters than is common with the board game version, though of course it is physically possible to play the board game with a very large number of counters. This shape is then mapped onto a grid to produce sets of squares which are then subjected to GoL rules. This is interesting for a number of reasons. Different scribbles are analog forms, each potentially different. However, the difference which will make a difference in the evolution of the form will not be evident in the initial conditions. This is one of the features of what Lorenz called “sensitivity to initial conditions” in chaotic systems (Lorenz, 1995). In symbiopoiesis theory, these larger shapes could be seen as superorganism which are not viable unless they have an internal structure
2. The complex form produced by the scribble quickly decomposes. Many of its squares die, leaving blank spaces. The remaining forms evolve into separate GoL shapes, each of which evolves following its own trajectory, or interferes with and is interfered by a closely adjacent form. That is, a shape is affected by others in its immediate environment, leading to a stable set of shapes, each of which is distinct from all others in the environment: an ecosystem. In symbiopoietic theory this is a compound form at a larger scale, produced as an unpredictable outcome of the same set of genetic and epigenetic rules (Figure 3).
3. However, this stability does not remain if any shape is a mover. Such forms collide with other shapes which otherwise were stable. GoL rules can lead to the destruction or transformation of either previous shape. Within the community formed out of the original scribble, there can be massive unpredictable changes. If this corresponds to the development process as described by Edelman, this implies that some events will be trans formative in the progress of development. In symbiopoietic theory there is an emphasis on homeostatic forms underpinning all organisms at all scales. This outcome is a reminder of the presence of pathogenic processes inherent in any complex biological system.
4. Sometimes movers leave the original space. They leave behind a stable, unchanging set of shapes. In ecological terms, this corresponds to one group in a community migrating to a new environment, leaving both the old and the new group less complex and less dynamic. In developmental terms, it would correspond to fission (cell or cell-collective) differentiation without any complementary regulatory process. However, in ecology and development there are no such empty spaces. In order to correspond more closely to this real-life situation it is necessary to have a higher level space containing more than one initial shape. This again models a process that symbiopoietic theory needs to take account of, where systemic processes lead to a loss of complexity in a holobiont.
5. When we used this feature we found that movers from different processes would collide with each other, or with stable shapes in other groupings. The outcomes of these collisions were unpredictable, though they followed from the simple rules of GoL. That is, phenomena at the developmental and ecological levels were produced by homologous processes, as in the rules of GoL, yet they also had distinctive emergent properties appropriate to their level.

## Discussion

GoL is a structure of rules organized as the interaction of two sets, existential rules and conditional rules, which correspond to a set of rules combining genetic and epigenetic forces and actions. This is the abstract specification we term the epigenetic principle, as it applies in biological systems.

Following Turing's work we note that both chemical and mechanical processes interact in epigenetic processes, and can be described as cellular automatons in GoL applications. But the same principles also apply to Von Neumann's cellular automatons, which can also be modeled in GoL-derived programs.

This set of relationships can be described as a relation of self-similarity between the three pairs of rules: existential = conditional: genetic = epigenetic: chemical = mechanical. The cellular automaton theory of GoL is what holds them together. Yet the relationship of self-similarity between them, and the fact that they operate on different scales, suggests that we look for underlying fractal theory (Mandelbrot, 1983).

The possibility of an underlying fractality is also suggested when we consider the different biological scales that GoL can be applied to. Conway's initial proposal seemed to reference ecology and the formation of groups or communities. But the game does not play out any differently if we suppose that groups of counters represent individual cells within a developing organism, cells in functional groups, individual organisms in their environment, or the evolution of groups, i.e., communities or societies.

GoL is scale invariant, so that the Game can be used about structures at different levels, from the development of cells and organisms to the evolution of communities and ecologies Symbiopoietic theory also has notions of scale built in, between different components of a superorganism which usually have different evolutionary histories and different scales of organization.

It would be interesting to investigate homologies across all these levels as produced by GoL. As we said at the outset, the generation of interesting hypotheses to test is a major benefit of the method of computer simulations we explore in this article.

Evo-devo is a framework which claims biologically based similarities between phylogenesis and ontogenesis, evolution and development, and mutual relations between the two levels. We can reframe these two terms and their binary relationship in a multiscalar structure in which both terms refer to structures with many levels linked by self-similarity.

A study of pigment patterning across a range of different lineages (Caballero et al., 2012) used a version of Turing's model to show how a combination of chemical and mechanical processes in the epigenetic environment could produce a wide variety of similar forms across widely separated species.

This work showed that only a small number of genes may have been involved in the organization of these morphogenetic processes, acting relatively early in the evolutionary process, actualized by a wide range of epigenetic mechanisms, chemical and mechanical. Self-similarity of patterns is found within a species (e.g., patterns of snakes, tigers, zebra-fish) and also across these species and families, a multilevel fractality which links development and evolution.

We use this instance to bring out both the limits and the value within those limits of a heuristic use of a computer simulation game like GoL. Because of its simplified parameters, GoL can be applied to many different biological levels and processes. On their own these resemblances may be only artifacts of the range of application. But that same range may also allow real but remote and unexpected resemblances to be represented, where they can be investigated in theory (e.g., in this case evo-devo, ecology and development) and through empirical research (e.g., in this case development of patterns in different lineages). Symbiopoietic phenomena similarly cross all phyla, and computer simulations like GoL may prove heuristically valuable in this field also.

We can model the potential value of a computer simulation game like GoL in terms of an epigenetic landscape. Without reference to biological and mathematical data, GoL remains a mere diversion. As a space in which creative scientific processes take place, biological and mathematical data can pass through a game-space as constituted by GoL, in which some ideas and facts are switched off and others are switched on, allowing new connections and new hypotheses to emerge. Those hypotheses do not become scientific until the normal painstaking work of science has been done. Yet without heuristic processes like GoL simulations, science may fail to test and find the new discoveries that have always been the greatest vindication of science.

## Author contributions

LC, BH, and SH have made substantial contributions to the conception and design of the work; SH worked with the simulations that is the base for the analysis and the illustrations. LC, BH, and SH participated in the interpretation for the work, which was done by LC, BH, and SH. LC, BH, and SH drafted different sections of the paper and all did the critical revision of the important intellectual content. LC, BH, and SH did the final approval of the version to be published and LC, BH, and SH are in agreement to be accountable for all aspects of the work in ensuring that questions related to the accuracy or integrity of any part of the work are appropriately investigated and resolved.

## Funding

Universidad Nacional Autonoma de México, UNAM, Centro de Ciencias de la Complejidad C3, UNAM, and Institute for Culture and Society, Western Sydney University, have supported the research activities of the Authors of this paper.

### Conflict of interest statement

The authors declare that the research was conducted in the absence of any commercial or financial relationships that could be construed as a potential conflict of interest.
